# Comparison of Immunoassay Results near Cutoff Values with LC-MS/MS in Individuals with Substance Use Disorder Under Probation

**DOI:** 10.3390/metabo16090651

**Published:** 2026-09-04

**Authors:** Ugur Fahri Yurekli, Ozlem Orer Beginoglu

**Affiliations:** 1 Department of Biochemistry, University of Health Sciences, Şanlıurfa Mehmet Akif İnan Training and Research Hospital, Şanlıurfa 63300, Türkiye; 2 Department of Psychiatry, University of Health Sciences, Şanlıurfa Mehmet Akif İnan Training and Research Hospital, Şanlıurfa 63300, Türkiye; ozlembeginoglu@gmail.com

**Keywords:** immunoassay, LC-MS/MS, urine drug testing, probation, cutoff value, forensic toxicology

## Abstract

**Highlights:**

**What are the main findings?**

- Agreement between immunoassay screening and LC–MS/MS confirmation varied markedly across analyte groups in urine samples with results near assay cutoff values.
- Immunoassays produced notable false-positive findings for amphetamines and opioids and false-negative findings for cannabinoids and opioids.

**What are the implications of the main findings?**

- Immunoassay results close to cutoff values should be interpreted cautiously because analytical performance is strongly analyte-dependent.
- LC–MS/MS confirmation is important for reliable clinical and forensic interpretation, particularly in probation monitoring where test results may have legal consequences.

**Abstract:**

**Background:** This cross-sectional study compared immunoassay urine drug screening with LC–MS/MS confirmation in individuals with substance use disorder undergoing probation monitoring, focusing on samples with results close to assay cutoff values. **Methods:** The study was conducted between January and March 2026 in Şanlıurfa, Türkiye, and included 110 individuals followed at an Addiction Treatment Center (AMATEM). Urine samples with at least one analyte result within ±20% of the cutoff value were analyzed. Immunoassay screening was performed using a Beckman Coulter AU480 analyzer, whereas confirmation was performed by LC–MS/MS using a SCIEX Triple Quad 5500+ system. Agreement was evaluated using Cohen’s kappa and McNemar’s test, while diagnostic performance was assessed using 2 × 2 contingency tables. **Results:** Agreement varied substantially among analyte groups. Buprenorphine showed the highest agreement (κ = 0.47), followed by amphetamines (κ = 0.33), whereas opioids showed very low agreement (κ = 0.081). Benzodiazepines, cocaine, and cannabinoids showed no meaningful agreement; however, benzodiazepine and cocaine findings were limited by very low numbers of positive cases. Immunoassay screening produced notable false-positive results for amphetamines and opioids and false-negative results for cannabinoids and opioids. **Conclusions:** Immunoassay performance was analyte-dependent and appeared limited in samples with results close to cutoff values. LC–MS/MS confirmation remains important for reliable toxicological interpretation in probation monitoring settings.

## 1. Introduction

Substance use disorder has become an increasingly significant public health problem from both clinical and forensic perspectives, particularly within probation systems, where regular toxicological monitoring of individuals is critically important. Urine-based screening tests are the most commonly utilized methods in this context due to their rapid turnaround time, ease of application, and low cost [1,2]. However, these assays may produce false-positive or false-negative results, especially at concentrations close to established cut-off values, due to their limited specificity [3,4]. In forensic toxicology practice, even minor analytical inaccuracies may result in substantial legal and social consequences, including inappropriate judicial sanctions or a misinterpretation of abstinence status. Therefore, analytical reliability has become a critical issue in drug testing programs conducted under legal supervision.

Although immunoassay methods allow rapid evaluation of large sample populations, cross-reactivity and metabolite interactions may compromise their reliability, particularly in borderline measurements near the cutoff threshold. Structurally similar compounds, prescription medications, endogenous metabolites, and emerging psychoactive substances can interfere with antibody-based detection systems and produce misleading results. This limitation can lead to misclassification with substantial implications for forensic decision-making processes. Recent studies have clearly demonstrated the need for confirmatory testing for immunoassay-based results, especially for samples with concentrations close to the cutoff range [5]. Furthermore, studies that evaluate urine drug testing methodologies have reported that confirmation significantly improves diagnostic accuracy and minimizes analytical uncertainty in forensic cases [6,7].

Liquid chromatography tandem mass spectrometry (LC-MS/MS) is considered the reference confirmatory method in toxicological analysis due to its high sensitivity and specificity [8]. The technique is increasingly utilized in clinical and forensic toxicology due to its ability to reliably quantify at low detection limits and perform simultaneous multi-analyte analysis [8]. Furthermore, LC-MS/MS-based methods provide an important advantage in modern toxicological investigations by allowing the detection of a broad spectrum of substances, including new psychoactive substances [9]. Recent analytical developments have also demonstrated the utility of high-throughput LC-MS/MS platforms for routine urine drug testing and large-scale forensic screening applications [10].

In addition to improving analytical sensitivity, LC-MS/MS substantially enhances selectivity through multiple reaction monitoring (MRM) transitions, thereby reducing interference from structurally related compounds. This capability is particularly important in probation populations where polysubstance use and concomitant exposure are common. Previous studies have demonstrated that LC-MS/MS provides greater specificity and accuracy than immunoassay screening tests and significantly reduces false-positive rates, particularly in cases involving multiple substance exposures. Consequently, the importance of LC-MS/MS confirmation has become increasingly evident to obtain reliable results in forensic settings [8,11]. Furthermore, recent forensic investigations have emphasized that comprehensive LC-MS/MS screening strategies allow for a more accurate identification of conventional drugs and newly emerging psychoactive compounds that may not be adequately detected by traditional immunoassays [12,13].

Another important challenge in urine toxicology analysis is the possibility of specimen adulteration or dilution intended to mask the use of substances. Advanced mass spectrometric techniques have recently shown promising performance in the identification of chemically adulterated urine specimens and in improving the reliability of toxicological interpretation [14]. These developments further support the increasing integration of mass spectrometry-based approaches into forensic toxicology laboratories around the world.

In probation practices, accurate and reliable determination of substance use status plays a crucial role in both judicial and rehabilitation processes. Reliable toxicological findings are essential not only for legal monitoring, but also to evaluate treatment adherence, relapse risk, and rehabilitation outcomes. Therefore, the necessity of confirming immunoassay results, particularly those near the cut-off threshold, using LC-MS/MS has been increasingly emphasized.

Although the general limitations of immunoassay screening and the value of LC–MS/MS confirmation are well established [4,6,7,15], previous studies have specifically evaluated immunoassay performance using challenging specimen sets enriched around screening cutoffs, demonstrating the analytical importance of results close to the decision threshold [16,17]. However, these studies used different approaches to select or define near-cutoff specimens and did not establish a universally accepted numerical interval. Furthermore, many previous comparisons evaluated overall assay performance, method validation, or unselected clinical and forensic samples. In contrast, the present study examined a diagnostically challenging cohort in which at least one immunoassay result was within ±20% of the relevant cutoff. This interval was prespecified as a pragmatic, study-specific definition rather than as a regulatory or universally standardized threshold. Such a targeted approach is particularly relevant in probation monitoring, where borderline results may influence both judicial decisions and rehabilitation processes.

Accordingly, this study aimed to evaluate the analyte-specific agreement and diagnostic performance of immunoassay screening compared with LC–MS/MS confirmation in individuals undergoing probation monitoring, specifically within a cohort selected on the basis of near-cutoff results. The study was designed to characterize false-positive and false-negative patterns in this analytically uncertain interval rather than to reassess the general superiority of LC–MS/MS over immunoassay screening.

## 2. Materials and Methods

### 2.1. Study Design and Participants

This retrospective cross-sectional method-comparison study included 110 individuals who presented to the Alcohol and Substance Addiction Treatment Center (AMATEM) in Şanlıurfa between January and March 2026 under probation supervision.

Individuals were eligible if immunoassay screening and LC–MS/MS confirmation were performed on the same urine specimen and at least one numerical semi-quantitative immunoassay drug-class result was within ±20% of the corresponding immunoassay screening cutoff. LC–MS/MS confirmation cutoffs were not used for specimen selection. The near-cutoff interval was defined a priori as an immunoassay result between 80% and 120% of the relevant screening cutoff. This pragmatic interval was selected to capture results close to the analytical decision threshold while retaining an adequate number of specimens; it was not based on a regulatory requirement, manufacturer-mandated range, or uniform estimate of analytical imprecision.

**Figure 1:**
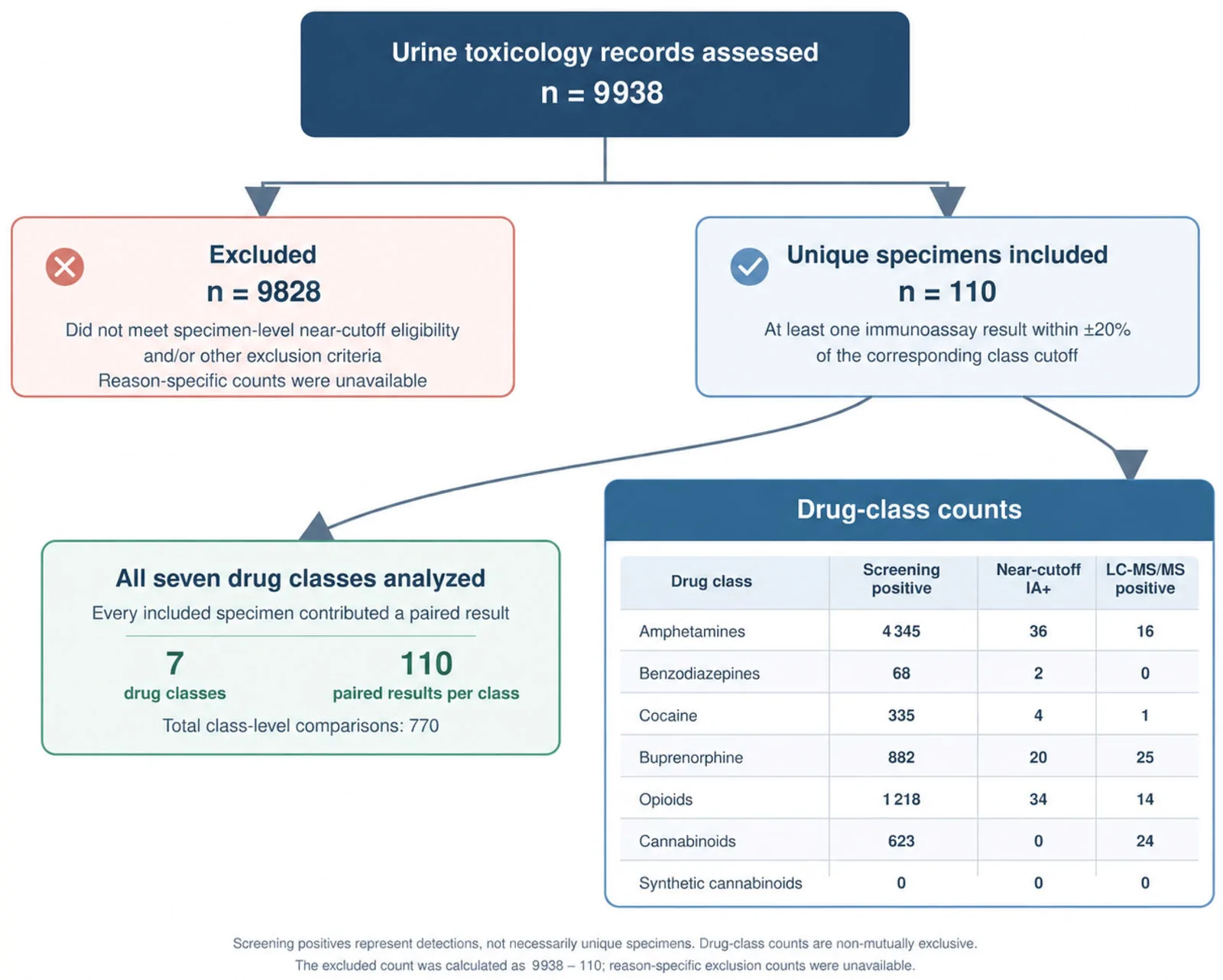
Flowchart of specimen selection and drug-class analyses. The ±20% eligibility criterion was applied to the immunoassay screening cutoff at the specimen level. Once a specimen met this criterion for at least one drug class, paired immunoassay and LC–MS/MS results for all seven drug classes were included in the analysis (n = 110 specimens for each drug class).

Eligibility was determined at the specimen level. If at least one immunoassay drug-class result met the near-cutoff criterion, paired immunoassay and LC–MS/MS results for all seven evaluated drug classes were analyzed in that specimen. Consequently, each drug-class analysis included the same 110 specimens, as summarized in Figure 1.

Specimens originating outside the AMATEM outpatient clinic, specimens with insufficient volume or documented preanalytical problems, analyses affected by instrument malfunction or quality-control failure, and repeated specimens from the same individual were excluded. For repeated submissions, only the first eligible specimen was included. Of the participants, 7 were women (6.4%) and 103 were men (93.6%), with a median age of 31.0 years (range: 17–61 years).

### 2.2. Specimen Collection

Urine specimens were collected under supervised conditions in the presence of security personnel in accordance with the 2025 Ministry of Health directive on the analysis of addictive substances. However, urine creatinine, specific gravity, pH, oxidizing adulterants, and other specimen-validity markers were not systematically measured or retrievable from the retrospective laboratory records. Therefore, specimens were not included or excluded on the basis of these parameters. Supervised collection was intended to reduce substitution and deliberate adulteration but did not provide analytical confirmation of specimen validity.

### 2.3. Immunoassay and LC–MS/MS Analyses

The comparative analyses included seven drug classes: amphetamines, benzodiazepines, cocaine, buprenorphine-related compounds, opioids, cannabinoids, and synthetic cannabinoids.

Immunoassay screening was performed on a Beckman Coulter AU480 automated analyzer (Beckman Coulter, Brea, CA, USA) using commercial Lin-Zhi International Inc. reagents (San Jose, CA, USA) in accordance with the manufacturer’s instructions. Numerical semi-quantitative results were classified as positive or negative using predefined screening cutoffs.

The Lin-Zhi buprenorphine immunoassay is calibrated against norbuprenorphine. According to the manufacturer, unconjugated buprenorphine shows approximately 101% cross-reactivity with norbuprenorphine, whereas cross-reactivity with buprenorphine-glucuronide and norbuprenorphine-glucuronide is approximately 0.1% and 0.9%, respectively. Thus, the assay detects unconjugated buprenorphine and norbuprenorphine but has limited sensitivity to their glucuronidated metabolites.

Confirmatory analysis was performed using a SCIEX Triple Quad 5500+ LC–MS/MS system (SCIEX, Framingham, MA, USA) and a multianalyte toxicology kit manufactured by Obikrom (Ankara, Türkiye). After centrifugation, 100 µL of urine, 20 µL of internal-standard solution, and 400 µL of diluent were mixed and vortexed for 1 min before injection. The analyte-specific composition of the manufacturer-supplied internal-standard reagent was not disclosed in the available Rev.08 instructions for use.

For THC-COOH confirmation, urine specimens were incubated with the hydrolysis enzyme reagent at 60 °C for 1 h. Hydrolysis efficiency was not independently verified using paired hydrolyzed and non-hydrolyzed specimens or glucuronidated THC-COOH control materials.

The LC–MS/MS panel included the seven evaluated drug classes and several additional drugs and metabolites. Detailed target analytes, confirmation cutoffs, and limits of quantification are presented in Supplementary Table S1. For drug-class comparisons, LC–MS/MS classification was considered positive when at least one analyte assigned to the corresponding drug class met its analyte-specific confirmation cutoff. Buprenorphine and norbuprenorphine were measured separately using their respective confirmation cutoffs; the buprenorphine-related class was considered positive when either analyte met its confirmation criterion.

### 2.4. Calibration and Quality Control

Matrix-matched lyophilized human urine calibrators were used with a seven-point calibration model (C0–C6). Quality control was performed at three concentration levels (L1–L3) in a human urine matrix, and analytical runs were accepted when the results were within the manufacturer-defined ranges.

According to the manufacturer’s validation data, within-run precision ranged from 1.4% to 18.9% CV, between-run precision from 1.5% to 16.9% CV, maximum absolute analytical bias was 18.5%, and recovery ranged from 81.13% to 114.41%. Analyte-specific linearity, carry-over, LOD, LOQ, precision, bias, and recovery data are summarized in Supplementary Table S2.

Complete historical analyte-specific in-house estimates of precision, recovery, and bias were not available from the retrospective laboratory records. Therefore, these performance characteristics should be interpreted as manufacturer-generated validation data rather than as an independent full analytical validation performed by our laboratory.

LC–MS/MS was used as the reference confirmatory method for statistical comparisons; however, this does not imply that analytical measurement or classification error was impossible.

### 2.5. Statistical Analysis

Statistical analyses were performed using MedCalc Statistical Software version 20.100 (MedCalc Software Ltd., Ostend, Belgium). Agreement between immunoassay and LC–MS/MS results was assessed using Cohen’s kappa coefficient. Kappa values were interpreted as poor (0.00–0.20), fair (0.21–0.40), moderate (0.41–0.60), good (0.61–0.80), or very good (0.81–1.00). Negative values indicated agreement worse than chance. Estimates based on very few positive cases were interpreted cautiously.

Discordance between paired categorical results was evaluated using McNemar’s test. A two-sided *p*-value of <0.05 was considered statistically significant. Using LC–MS/MS as the reference method, 2 × 2 contingency tables were constructed to calculate sensitivity, specificity, positive predictive value, negative predictive value, and overall accuracy. A parameter was reported as not estimable when its denominator was zero. Estimates based on very small numbers of positive cases, particularly cocaine, were considered descriptive.

ROC analysis was not performed because the comparative results were binary and several drug classes contained very few or no positive cases. Because inclusion required at least one near-cutoff immunoassay result, the performance estimates apply specifically to this selected cohort and should not be generalized to an unselected routine-testing population.

## 3. Results

A total of 110 unique urine specimens obtained from individuals undergoing probation monitoring met the specimen-level inclusion criterion. Each included specimen had paired immunoassay and LC–MS/MS results available for all seven evaluated drug classes. Therefore, 110 paired observations were included in each drug-class analysis, irrespective of which drug-class result initially met the near-cutoff eligibility criterion. Immunoassay screening results were compared with LC-MS/MS confirmation results for amphetamines, benzodiazepines, cocaine, buprenorphine, opioids, cannabinoids, and synthetic cannabinoids. The agreement between the two methods and diagnostic performance parameters are presented in Table 1 and Table 2.

In the amphetamine group, 12 samples were positive by both methods, whereas 24 samples were positive only by immunoassay and 4 samples were positive only by LC-MS/MS. A fair level of agreement was observed between the two methods (κ = 0.33; 95% CI: 0.15–0.51). McNemar’s test indicated a statistically significant difference between the methods (*p* = 0.0002; Table 1). The sensitivity, specificity, positive predictive value (PPV), negative predictive value (NPV), and accuracy of immunoassay for amphetamines were 75.0%, 74.5%, 33.3%, 94.6%, and 74.5%, respectively (Table 2).

In the benzodiazepine group, no LC-MS/MS-positive sample was identified. Two samples were positive only by immunoassay, whereas 108 samples were negative by both methods. No meaningful agreement was observed between the methods (κ = 0.00), and McNemar’s test showed no statistically significant difference (*p* = 0.50; Table 1). Because there were no LC-MS/MS-positive cases, sensitivity could not be estimated. The specificity, PPV, NPV, and accuracy were 98.2%, 0.0%, 100.0%, and 98.2%, respectively. However, these estimates should be interpreted cautiously because of the absence of true positive cases (Table 2).

In the cocaine group, 1 sample was positive by both methods, while 3 samples were positive only by immunoassay. No sample was positive only by LC-MS/MS, and 106 samples were negative by both methods. The kappa value was 0.39, and the difference between the two methods was not statistically significant according to McNemar’s test (*p* = 0.25; Table 1). The sensitivity was calculated as 100.0%; however, this estimate was based on only one LC-MS/MS-positive case and should therefore be interpreted with caution. The specificity, PPV, NPV, and accuracy were 97.2%, 25.0%, 100.0%, and 97.3%, respectively (Table 2).

In the buprenorphine group, 13 samples were positive by both methods, whereas 7 samples were positive only by immunoassay and 12 samples were positive only by LC-MS/MS. Seventy-eight samples were negative by both methods. A moderate level of agreement was observed between immunoassay and LC-MS/MS (κ = 0.47; 95% CI: 0.27–0.67), and McNemar’s test showed no statistically significant difference between the methods (*p* = 0.36; Table 1). The sensitivity, specificity, PPV, NPV, and accuracy of immunoassay for buprenorphine were 52.0%, 91.8%, 65.0%, 86.7%, and 82.7%, respectively (Table 2).

In the opioid group, 6 samples were positive by both methods, whereas 28 samples were positive only by immunoassay and 8 samples were positive only by LC-MS/MS. Sixty-eight samples were negative by both methods. The agreement between the two methods was very low (κ = 0.081; 95% CI: −0.09 to 0.25). McNemar’s test indicated a statistically significant difference between the methods (*p* = 0.0012; Table 1). The sensitivity, specificity, PPV, NPV, and accuracy were 42.9%, 70.8%, 17.6%, 89.5%, and 67.3%, respectively (Table 2).

In the cannabinoid group, no sample was positive by immunoassay, whereas 24 samples were positive by LC-MS/MS. Eighty-six samples were negative by both methods. No agreement was observed between the methods (κ = 0.00), and McNemar’s test showed a statistically significant difference (*p* < 0.0001; Table 1). The sensitivity of immunoassay for cannabinoids was 0.0%, while specificity was 100.0%. PPV was not estimable because no immunoassay-positive result was obtained. The NPV and accuracy were 78.2% and 78.2%, respectively (Table 2).

For synthetic cannabinoids, no positive result was detected by either immunoassay or LC-MS/MS. Therefore, kappa and McNemar analyses were not applicable. Although specificity, NPV, and accuracy were numerically 100.0%, these diagnostic performance metrics are of limited interpretability because there were no positive cases in this group (Table 1 and Table 2).

Overall, the highest agreement between immunoassay and LC–MS/MS was observed for buprenorphine, followed by amphetamines, whereas agreement was very low for opioids and absent for cannabinoids in this selected cohort. For benzodiazepines, cocaine, and synthetic cannabinoids, the absence or extremely low number of LC–MS/MS-positive cases precluded reliable assessment of agreement and diagnostic performance. Therefore, the numerical findings for these analytes should be regarded as exploratory and descriptive rather than as stable estimates of sensitivity, predictive values, or agreement. The diagnostic performance of immunoassay varied substantially among analyte groups, with particularly low sensitivity for cannabinoids and opioids, and limited PPV for amphetamines, cocaine, and opioids.

## 4. Discussion

In this study, immunoassay-based urine screening results were compared with LC-MS/MS confirmation results in individuals with substance use disorder undergoing probation monitoring, with particular emphasis on samples close to established cutoff values. The findings demonstrated that the agreement and diagnostic performance of immunoassay varied substantially across substance groups. This variation is clinically and forensically important because inaccurate classification of urine drug testing results near cutoff thresholds may directly affect probation monitoring, rehabilitation decisions, and judicial outcomes [18,19]. The recent literature has also emphasized that confirmatory testing is particularly important in drug testing workflows when presumptive screening results may have clinical, forensic, or legal consequences [3].

The principal contribution of the present study lies not in demonstrating the general superiority of LC–MS/MS confirmation, which has already been established, but in evaluating method discordance within a deliberately selected near-cutoff cohort. By restricting inclusion to specimens with at least one immunoassay result within ±20% of the relevant cutoff, the study focused on the interval in which analytical classification is most uncertain and the consequences of misclassification may be greatest. Moreover, the analyte-specific analysis showed that discordance was not uniform across drug classes: false-positive findings predominated for some analytes, whereas marked false-negative patterns were observed for others. These findings provide setting-specific evidence that borderline immunoassay results cannot be interpreted using a uniform approach across all analyte groups.

Agreement between immunoassay and LC-MS/MS ranged from poor to moderate across analyte groups. The highest agreement was observed for buprenorphine, which showed moderate agreement between the two methods. Amphetamines demonstrated fair agreement, whereas opioids showed very poor agreement. No meaningful agreement was observed for benzodiazepines, cocaine, and cannabinoids; however, the interpretation of benzodiazepine and cocaine results is limited by the extremely low number of positive cases. These findings suggest that the reliability of immunoassay screening is analyte-dependent and may be particularly vulnerable in near-cutoff samples [7,15,20].

For amphetamines, immunoassay showed fair agreement with LC-MS/MS and demonstrated relatively acceptable sensitivity and specificity compared with several other analyte groups. However, the positive predictive value was low, indicating that a substantial proportion of immunoassay-positive amphetamine results were not confirmed by LC-MS/MS. This finding highlights the potential risk of false-positive interpretation when immunoassay results are used alone, particularly in legally sensitive settings. Therefore, even for analytes with comparatively better agreement, confirmation by LC-MS/MS remains important when results have forensic consequences [5,18,19]. Modern LC-MS/MS-based toxicology methods provide high analytical specificity and allow simultaneous detection of multiple drugs and metabolites, which is advantageous in forensic and clinical toxicology settings [5,13].

The buprenorphine group showed moderate agreement between immunoassay and LC-MS/MS, with higher specificity than sensitivity. Although the revised analysis demonstrated better agreement than initially observed, false-negative results were still present. A more detailed evaluation of LC-MS/MS-positive buprenorphine-related findings showed that only 2 individuals had buprenorphine concentrations above the confirmation cutoff, whereas 18 individuals were positive for norbuprenorphine. Although these findings indicate a predominance of norbuprenorphine among the LC–MS/MS-positive results, this pattern should not be interpreted as evidence that the immunoassay has limited recognition of norbuprenorphine. The assay is calibrated against norbuprenorphine and shows approximately equivalent reactivity to unconjugated buprenorphine. Therefore, the observed discordance may instead reflect differences between the combined immunoassay response and the separate analyte-specific classification used by LC–MS/MS, concentrations close to the respective decision thresholds, and the minimal cross-reactivity of the immunoassay with glucuronidated metabolites. Because complete quantitative concentration and conjugation-profile data were unavailable for all discordant specimens, the precise contribution of these factors could not be determined.

In the opioid group, both false-positive and false-negative results were observed, and agreement between immunoassay and LC-MS/MS was very low. The low positive predictive value indicates that many immunoassay-positive opioid results were not confirmed by LC-MS/MS. This may be related to antibody cross-reactivity with structurally related compounds or differences in the opioid metabolites targeted by the two methods. Conversely, false-negative results may reflect limited immunoassay sensitivity for specific opioid metabolites or concentrations close to the cutoff threshold. These findings support the need for confirmatory testing in opioid screening, especially in forensic and probation settings where misclassification may have serious consequences [3,5,18]. Recent multi-analyte LC-MS/MS methods have been developed to address the analytical challenges posed by structurally diverse drug classes and emerging psychoactive substances [8,13].

The cannabinoid group represented one of the clearest examples of discordance. No cannabinoid-positive result was detected by immunoassay, whereas 24 samples were positive by LC-MS/MS. Consequently, immunoassay sensitivity for cannabinoids was 0.0%, while specificity was 100.0%. This pattern indicates that the immunoassay failed to identify LC-MS/MS-confirmed cannabinoid-positive samples in this near-cutoff population. Several factors may explain this finding, including differences in cutoff concentrations, antibody recognition of cannabinoid metabolites, concentrations close to the immunoassay decision threshold, and pre-analytical factors such as urine dilution. In particular, dilution could decrease the apparent THC-COOH concentration below the immunoassay cutoff while the analyte remains detectable by the more analytically sensitive LC–MS/MS method. However, urine creatinine, specific gravity, pH, oxidizing adulterants, and other specimen validity markers were not systematically available. Therefore, dilution, adulteration, substitution, or another specimen-integrity problem cannot be objectively excluded, and their potential contribution to the 24 immunoassay-negative but LC–MS/MS-positive cannabinoid findings could not be quantified. Accordingly, this discordance should not be attributed solely to limited immunoassay performance. In addition, the efficiency of the enzymatic hydrolysis step used for LC–MS/MS confirmation of THC-COOH was not independently assessed. Incomplete hydrolysis could reduce the measured concentration of free THC-COOH and potentially result in false-negative LC–MS/MS findings. However, it would not explain the immunoassay-negative samples that were positive by LC–MS/MS. If incomplete hydrolysis occurred, it would more likely have reduced the number of LC–MS/MS-positive cannabinoid samples and attenuated, rather than exaggerated, the observed discordance between the two methods. This limitation should be considered when interpreting the cannabinoid findings [3,12,13].

In the benzodiazepine group, no LC-MS/MS-positive sample was identified. Therefore, sensitivity could not be estimated, and the diagnostic performance of immunoassay for benzodiazepines cannot be reliably inferred from the present data. Similarly, the cocaine group included only one LC-MS/MS-positive sample. Although sensitivity was numerically calculated as 100.0%, this estimate is based on a single positive case and lacks statistical robustness. With only one LC–MS/MS-positive cocaine specimen, the classification of a single additional sample would markedly alter the sensitivity estimate. Therefore, the reported sensitivity of 100.0% should not be interpreted as evidence of high immunoassay sensitivity for cocaine. Similarly, the absence of LC–MS/MS-positive benzodiazepine samples means that the study provides information only on negative-case concordance and false-positive screening findings, not on the ability of the immunoassay to detect benzodiazepine-positive specimens. Cohen’s kappa is also unstable under these very low-prevalence conditions and should not be considered a robust measure of agreement for either analyte. Therefore, findings for benzodiazepines and cocaine should be considered exploratory and interpreted with extreme caution. Larger studies with sufficient numbers of positive cases are required to provide reliable estimates for these analytes [10,20]. High-throughput urine drug testing studies also emphasize the importance of sufficient analyte coverage and validated confirmation methods when interpreting screening performance [10].

No positive result was detected by either method for synthetic cannabinoids. Although specificity, negative predictive value, and accuracy were numerically 100.0%, these values reflect only concordant negative findings and do not demonstrate that the immunoassay can detect synthetic cannabinoid-positive specimens. Because no positive specimen was identified by either method, sensitivity, positive predictive value, and meaningful between-method agreement could not be assessed. Accordingly, these findings are descriptive and exploratory and do not permit any conclusion regarding the diagnostic performance of the immunoassay for synthetic cannabinoids. Therefore, the present data do not allow a meaningful conclusion regarding the diagnostic performance of immunoassay for synthetic cannabinoids. This is particularly important because synthetic cannabinoids and other new psychoactive substances are structurally heterogeneous, and routine screening panels may not adequately cover newly emerging compounds [3,13]. The continuously evolving spectrum of new psychoactive substances is a well-recognized challenge for conventional drug testing strategies and often requires expanded mass spectrometry-based approaches [3].

Several limitations should be considered when interpreting the present findings. First, this was a single-center study with a limited sample size. More importantly, the inclusion criterion deliberately enriched the cohort with analytically challenging specimens by requiring at least one immunoassay result to be within ±20% of the relevant cutoff. This selection strategy introduced spectrum bias because the distribution of positive, negative, and discordant findings in this cohort is unlikely to reflect that of an unselected routine urine drug-testing population. Accordingly, the reported sensitivity, specificity, positive predictive value, negative predictive value, and accuracy estimates represent conditional performance within this selected near-cutoff cohort and should not be interpreted as measures of overall immunoassay performance in routine practice. PPV and NPV are particularly dependent on the prevalence and spectrum of the population evaluated and may therefore differ substantially in an unselected testing population. Second, the number of LC–MS/MS-positive cases was zero or extremely low for benzodiazepines, cocaine, and synthetic cannabinoids. Under these conditions, sensitivity cannot be estimated when no reference-positive case is present, while an estimate based on a single positive case is statistically unstable. PPV and Cohen’s kappa are likewise sensitive to extremely low prevalence and sparse cell counts. Consequently, the numerical findings for these analytes should be regarded as exploratory and descriptive rather than as reliable estimates of diagnostic performance. Third, because immunoassay and LC–MS/MS results were evaluated as binary positive/negative outcomes, receiver operating characteristic curve analysis was not performed. Instead, diagnostic performance was assessed using contingency table-based parameters. Fourth, objective specimen validity data, including urine creatinine concentration, specific gravity, pH, oxidizing adulterants, and other adulteration markers, were not systematically available. Although supervised collection may have reduced the risk of substitution or deliberate adulteration, it could not exclude dilution or other specimen-integrity problems. This limitation is particularly relevant to the cannabinoid group because dilution may have reduced THC-COOH concentrations below the immunoassay cutoff while remaining detectable by LC–MS/MS. Therefore, the contribution of specimen dilution or adulteration to the observed discordance could not be determined. Therefore, the mechanisms underlying some false-positive and false-negative results could not be fully clarified. Fifth, although manufacturer-reported validation characteristics were available, complete raw validation data and independent retrospective in-house analyte-specific estimates of precision, recovery, and analytical bias were unavailable. The analyte-specific composition of the manufacturer-supplied internal-standard reagent was also not disclosed. Therefore, potential LC–MS/MS measurement or classification error, particularly for concentrations close to the confirmation cutoffs, cannot be completely excluded. Because LC–MS/MS served as the reference method, such errors could have affected the classification of true-positive, false-positive, true-negative, and false-negative results and the resulting diagnostic performance estimates. Finally, the efficiency of the enzymatic hydrolysis procedure used for THC-COOH confirmation was not independently validated using glucuronidated control materials. Consequently, incomplete hydrolysis and a resulting underestimation of LC–MS/MS cannabinoid positivity cannot be completely excluded.

Within this deliberately selected near-cutoff cohort, immunoassay screening performance differed substantially among substance groups, and both the direction and magnitude of discordance varied by analyte. These findings characterize performance specifically within the analytically uncertain interval around the cutoff and should not be extrapolated to unselected routine urine drug-testing populations. The risk of false-positive and false-negative results was particularly evident for opioids and cannabinoids, whereas benzodiazepine, cocaine, and synthetic cannabinoid results were limited by low or absent positive case numbers. LC-MS/MS confirmation remains essential for accurate interpretation of urine drug testing results in legally sensitive settings, particularly when immunoassay results are close to cutoff values or when discordant findings may have judicial consequences [5,10,13]. However, because of the spectrum bias introduced by the selection strategy, the single-center design, and the limited number of positive cases for several analytes, the present findings should be interpreted cautiously. Studies using consecutive or randomly selected routine specimens are required to determine overall immunoassay performance and the generalizability of these findings.

## 5. Conclusions

This study evaluated the comparative performance of immunoassay urine drug screening and LC-MS/MS confirmation in individuals with substance use disorder undergoing probation monitoring, with particular emphasis on samples close to established cutoff values. The findings demonstrated that immunoassay performance varied considerably among substance groups and that agreement with LC-MS/MS ranged from poor to moderate. These results are clinically and forensically relevant because inaccurate classification of urine drug testing results may affect probation monitoring, rehabilitation decisions, and judicial outcomes.

Among the evaluated analyte groups, buprenorphine showed the highest agreement between immunoassay and LC-MS/MS, followed by amphetamines. However, buprenorphine still demonstrated limited sensitivity, indicating that some LC-MS/MS-confirmed positive cases were not detected by immunoassay. Amphetamines showed fair agreement but a low positive predictive value, suggesting a risk of false-positive screening results. Opioids showed very low agreement, with both false-positive and false-negative findings, whereas cannabinoids demonstrated a marked false-negative pattern, as no immunoassay-positive result was detected despite LC-MS/MS-confirmed positive cases. For benzodiazepines, cocaine, and synthetic cannabinoids, the absence or extremely low number of LC–MS/MS-positive cases precluded reliable estimation of sensitivity, predictive values, and agreement. Findings for these analytes should therefore be considered exploratory observations and should not be interpreted as evidence of either adequate or inadequate immunoassay performance.

Within this selected near-cutoff cohort, the combined evaluation of agreement and contingency table-based performance parameters showed that immunoassay results were vulnerable to analyte-specific misclassification. These findings apply specifically to specimens within the analytically uncertain interval around the cutoff and do not represent the overall diagnostic performance of immunoassays in routine urine drug-testing populations. LC–MS/MS confirmation remains important when near-cutoff findings may influence clinical, forensic, or probation-related decisions.

In conclusion, the present findings support cautious interpretation and LC–MS/MS confirmation of immunoassay results within the near-cutoff interval when toxicological findings may have legal or probation-related consequences. Because the cohort was deliberately enriched for near-cutoff specimens, the reported diagnostic performance estimates should not be generalized to routine urine drug-testing populations. Future multicenter studies using consecutive or randomly selected routine specimens are needed to determine overall immunoassay performance, while targeted near-cutoff studies may help further define analyte-specific sources of discordance.

## Figures and Tables

**Table 1:** Comparison of Immunoassay and LC-MS/MS results.

Analyte			Immunoassay		Kappa (95% CI)	McNemar *p*-Value
			Positive	Negative		
**Amphetamines**	**LC-MS/MS**	Positive	12	24	0.33 (0.15–0.51)	0.0002
		Negative	4	70		
Benzodiazepines *		Positive	0	2	0.00	0.50
		Negative	0	108		
Cocaine *		Positive	1	3	0.39	0.25
		Negative	0	106		
**Buprenorphine**		Positive	13	7	0.47 (0.27 to 0.67)	0.36
		Negative	12	78		
**Opioids**		Positive	6	28	0.085 (−0.09 to 0.25)	0.0012
		Negative	8	68		
**Cannabinoids**		Positive	0	0	0.00	<0.0001
		Negative	24	86		
**Synthetic Cannabinoids**		Positive	0	0	–	–
		Negative	0	110		

CI: confidence interval; Kappa: Cohen Kappa; *p*-value: McNemar test; * Because of the absence or extremely low number of LC–MS/MS-positive cases, kappa estimates for benzodiazepines and cocaine are statistically unstable and should be regarded as exploratory rather than as reliable measures of agreement. Kappa was not applicable for synthetic cannabinoids because no positive result was detected by either method.

**Table 2:** Diagnostic Performance of Immunoassay Screening Compared with LC-MS/MS Confirmation for Each Analyte.

Analyte	FP	FN	TP	TN	Sensitivity (%)	Specificity (%)	PPV (%)	NPV (%)	Accuracy (%)
Amphetamines	24	4	12	70	75.0	74.5	33.3	94.6	74.5
Benzodiazepines	2	0	0	108	NE	98.2	0.0	100.0	98.2
Cocaine	3	0	1	106	100.0 *	97.2	25.0	100.0	97.3
Buprenorphine	7	12	13	78	52.0	91.8	65.0	86.7	82.7
Opioids	28	8	6	68	42.9	70.8	17.6	89.5	67.3
Cannabinoids	0	24	0	86	0.0	100.0	NE	78.2	78.2
Synthetic cannabinoids	0	0	0	110	NE	100.0	NE	100.0	100.0 ^†^

FP: false positive; FN: false negative; TP: true positive; TN: true negative; PPV: positive predictive value; NPV: negative predictive value; NE: not estimable. LC–MS/MS was accepted as the reference method. Diagnostic performance estimates for benzodiazepines, cocaine, and synthetic cannabinoids should be regarded as descriptive and exploratory because of the absence or extremely low number of LC–MS/MS-positive cases. Sensitivity was not estimable for analytes with no LC–MS/MS-positive specimens. * The cocaine sensitivity estimate was based on only one LC–MS/MS-positive specimen and is therefore statistically unstable. ^†^ For synthetic cannabinoids, the numerically high specificity, NPV, and accuracy reflect only concordant negative findings and do not indicate the ability of the immunoassay to detect positive specimens.

## Data Availability

The data supporting the findings of this study are not publicly available due to ethical and patient privacy restrictions. De-identified data may be provided by the corresponding author upon reasonable request and with appropriate institutional approvals.

## Supplementary Materials

The following supporting information can be downloaded at: https://www.mdpi.com/article/10.3390/metabo16090651/s1, Table S1: Target analytes, immunoassay screening cutoffs, LC-MS/MS reporting thresholds, LOD and LOQ values; and Table S2: Quality-control levels and manufacturer-defined acceptable ranges.
