# Supplementary material for: Comparison of Immunoassay Results near Cutoff Values with LC-MS/MS in Individuals with Substance Use Disorder Under Probation
Source: Metabolites. 2026 Sep 4;16(9):651. doi: 10.3390/metabo16090651 (PMC13609008; doi:10.3390/metabo16090651)
Supplement: Supplementary file 1 [file metabolites-16-00651-s001.zip › metabolites-4466016-supplementary.pdf]

Supplementary File

Comparison of Immunoassay Results Near Cutoff Values with LC-MS/MS in Individuals with Substance Use Disorder Under Probation

Ugur Fahri Yurekli [1] *, and Ozlem Orer Beginoglu [2]

[1] Department of Biochemistry, University of Health Sciences, Şanlıurfa Mehmet Akif İnan Training and Research Hospital, Şanlıurfa 63300, Türkiye; ugurrllab@gmail.com

[2] Department of Psychiatry, University of Health Sciences, Şanlıurfa Mehmet Akif İnan Training and Re-search Hospital, Şanlıurfa 63300, Türkiye; ozlembeginoglu@gmail.com

* Correspondence: ugurrllab@gmail.com; Tel: +90532 777 9399

LC-MS/MS analytical conditions, calibration, quality control and validation details

Supplementary Table S1. Target analytes, immunoassay screening cutoffs, LC-MS/MS reporting thresholds, LOD and LOQ values

LC-MS/MS reporting thresholds represent the laboratory interpretation thresholds used for final qualitative classification. LOD and LOQ values are taken from the manufacturer validation report when available. N/A indicates that the value was not available in the accessible validation documentation.

**Drug class**	**Analyte / metabolite**	**Immunoassay screening cutoff (ng/mL)**	**LC-MS/MS reporting threshold (ng/mL)**	**LOD (ng/mL)**	**LOQ (ng/mL)**
Amphetamines	Amphetamine	<500	200	0.26	0.78
Amphetamines	Methamphetamine	<500	200	0.37	0.41
Amphetamines	MDMA	<500	200	0.09	0.37
Amphetamines	MDA	<500	200	0.96	1.77
Amphetamines	MDEA	<500	200	0.08	0.49
Amphetamines	Methylmethcathinone	<500	1	0.08	0.30
Amphetamines	Methcathinone	<500	5	0.31	0.49
Amphetamines	Cathinone	<500	5	0.14	0.23
Amphetamines	MBDB	<500	1	0.11	0.50
Cannabinoids	THC-COOH	<50	15	0.50	1.41
Cannabinoids	Delta-9 THC	<50	15	N/A	15
Cocaine	Benzoylecgonine	<150	100	1.10	2.61
Cocaine	Cocaine	<150	150	0.14	0.23
Cocaine	Norcocaine	<150	10	0.08	0.49
Cocaine	Cocaethylene	<150	1	0.18	0.28
Cocaine	Propylbenzoylecgonine	<150	1	N/A	N/A
Buprenorphine	Buprenorphine	<10	2	0.27	1.42
Buprenorphine	Norbuprenorphine	<10	2	0.52	1.43
Opioids	Codeine	<2000	300	0.20	0.70
Opioids	Morphine	<2000	300	0.20	0.64

Drug class	Analyte / metabolite	Immunoassay screening cutoff (ng/mL)	LC-MS/MS reporting threshold (ng/mL)	LOD (ng/mL)	LOQ (ng/mL)
Opioids	Morphine-3-Glucuronide	<2000	10	0.36	0.42
Opioids	Morphine-6-Glucuronide	<2000	10	0.67	0.94
Opioids	6-MAM	<2000	10	0.14	0.53
Opioids	Heroin	<2000	10	0.24	0.57
Opioids	Methadone	<2000	1	0.13	0.30
Opioids	EDDP	<2000	1	0.30	0.70
Opioids	Fentanyl	<2000	1	0.01	0.03
Opioids	Naloxone	<2000	1	0.17	0.46
Opioids	Naltrexone	<2000	1	0.30	0.91
Opioids	Norcodeine	<2000	10	0.28	0.66
Opioids	Normorphine	<2000	10	0.33	0.77
Opioids	Hydromorphone	<2000	100	0.12	0.27
Opioids	Hydrocodone	<2000	100	0.19	0.26
Opioids	Oxycodone	<2000	100	0.14	0.25
Opioids	Ethylmorphine	<2000	5	0.22	0.28
Opioids	Dihydrocodeine	<2000	10	0.33	0.47
Opioids	Meperidine	<2000	5	0.08	0.30
Opioids	Thebaine	<2000	5	0.11	0.12
Benzodiazepines	Alprazolam	<300	100	0.09	0.27
Benzodiazepines	Clonazepam	<300	100	0.55	0.87
Benzodiazepines	Diazepam	<300	100	0.21	0.37
Benzodiazepines	Flunitrazepam	<300	1	0.10	0.31
Benzodiazepines	Lorazepam	<300	100	1.06	3.28
Benzodiazepines	Oxazepam	<300	100	2.06	3.29
Benzodiazepines	Nitrazepam	<300	100	0.46	0.87
Benzodiazepines	Bromazepam	<300	100	0.50	0.91
Benzodiazepines	Flurazepam	<300	100	0.05	0.33
Benzodiazepines	Clobazam	<300	300	0.19	0.26
Benzodiazepines	Triazolam	<300	300	0.11	0.26
Benzodiazepines	7-Aminoclonazepam	<300	100	0.35	0.41
Benzodiazepines	Midazolam	<300	100	0.15	0.28
Benzodiazepines	Nordiazepam	<300	100	0.23	0.76
Synthetic Cannabinoids	AKB-48	<10	1	N/A	5
Synthetic Cannabinoids	5F-PB-22	<10	20	N/A	5
Synthetic Cannabinoids	4F-ABUTINACA-1	<10	5	N/A	N/A
Synthetic Cannabinoids	4F-ABUTINACA-N-(4-hydroxybutyl)	<10	5	N/A	N/A
Synthetic Cannabinoids	4F-MDMB-BUTINACA-M	<10	5	N/A	N/A
Synthetic Cannabinoids	4F-MDMB-Butica	<10	5	N/A	N/A
Synthetic Cannabinoids	4F-MDMB-Butica-N-(4-hydroxybutyl)-M	<10	5	N/A	N/A
Synthetic Cannabinoids	5F-Bzo-poxizid (MDA-19)	<10	5	N/A	N/A
Synthetic Cannabinoids	5F-ADB-PINACA	<10	5	N/A	N/A
Synthetic Cannabinoids	5F-ADBICA	<10	5	N/A	N/A
Synthetic Cannabinoids	5F-APICA	<10	5	N/A	N/A
Synthetic Cannabinoids	5F-APINACA N-(4-Hydroxypentyl)	<10	5	N/A	N/A
Synthetic Cannabinoids	5F-EMB-PICA	<10	5	N/A	N/A
Synthetic Cannabinoids	5F-MDMB-PICA-M7	<10	5	N/A	N/A
Synthetic Cannabinoids	5F-MDMB-PINACA-M2	<10	5	N/A	N/A
Synthetic Cannabinoids	5F-MDMB-PINACA-M7	<10	5	N/A	N/A
Synthetic Cannabinoids	5F-PB-22-3-Carboxyindole	<10	5	N/A	N/A
Synthetic Cannabinoids	AB-PINACA Pentanoic Acid	<10	5	N/A	N/A
Synthetic Cannabinoids	ADB-PINACA	<10	5	N/A	N/A
Synthetic Cannabinoids	ADB-PINACA Pentanoic Acid	<10	5	N/A	N/A
Synthetic Cannabinoids	ADB-HEXINACA	<10	5	N/A	N/A
Synthetic Cannabinoids	ADB-INACA	<10	5	N/A	N/A
Synthetic Cannabinoids	ADB-BUTINACA-N-(4-hydroxybutyl)-M	<10	5	N/A	N/A
Synthetic Cannabinoids	AM2201 N-(4-Hydroxypentyl)	<10	5	N/A	N/A
Synthetic Cannabinoids	BB-22	<10	5	N/A	N/A
Synthetic Cannabinoids	BB-22-3-Carboxyindole	<10	5	N/A	N/A
Synthetic Cannabinoids	MDA-19	<10	5	N/A	N/A
Synthetic Cannabinoids	MDMB-4en PINACA BAM	<10	5	N/A	N/A
Synthetic Cannabinoids	MDMB-4en PINACA BAM2	<10	5	N/A	N/A
Synthetic Cannabinoids	MMB-2201	<10	5	N/A	N/A
Synthetic Cannabinoids	NM-2201	<10	5	N/A	N/A
Synthetic Cannabinoids	PB-22-3-Carboxyindole	<10	5	N/A	N/A

Supplementary Table S2. Quality-control levels and manufacturer-defined acceptable ranges

Quality-control materials were urine-based controls (lot TS250709KL). Values are shown as mean (acceptable range), ng/mL. Representative grouped rows are shown for analytes sharing the same QC concentration scheme.

Analyte group / representative analytes	L1 mean (range), ng/mL	L2 mean (range), ng/mL	L3 mean (range), ng/mL
Amphetamine/Methamphetamine/MDMA/MDA/MDEA/MBDB and related cathinones	35.56 (28.45–42.67)	106.67 (85.34–128)	320.00 (256–384)
Codeine/Morphine/Dihydrocodeine/Norcodeine/BEG/Cocaine/EDDP/Ethylmorphine/Normorphine	17.78 (14.22–21.33)	53.33 (42.67–64)	160.00 (128–192)
6-MAM	8.89 (7.11–10.67)	26.67 (21.34–32.0)	80.00 (64–96)
Methadone/Oxycodone/Naltrexone/Naloxone/Benzodiazepines/Pseudoephedrine/Ketamine/Quetiapine/Ephedrine/Norpseudoephedrine	26.67 (21.34–32.0)	80.00 (64–96)	240.00 (192–288)
Buprenorphine/Norbuprenorphine	8.90 (7.12–10.68)	26.65 (21.32–31.98)	80.00 (64.0–96.0)
Fentanyl	0.89 (0.72–1.06)	2.67 (2.13–3.20)	8.00 (6.40–9.60)
